# Integrative multi-omics analysis identifies *GNAZ* and *SLC5A4* as critical regulators of gestation length in Large White pigs

**DOI:** 10.1186/s12711-026-01058-5

**Published:** 2026-06-10

**Authors:** Jin Zhou, Qingbo Zhao, Xiaowen Qian, Tao Zeng, Liming Xu, Qian Liu, Yanzhen Yin, Jinfeng Ma, Jianghui Yu, Zheng Jiang, Guankai Zhao, Ruihua Huang, Pinghua Li

**Affiliations:** 1 Sanya Institute of Nanjing Agricultural University, Sanya, 572024 People’s Republic of China; 2 Key Laboratory in Nanjing for Evaluation and Utilization of Pig Resources, Ministry of Agriculture and Rural Areas of China, Institute of Swine Science, Nanjing Agricultural University, Nanjing, 210095 People’s Republic of China; 3 Huaian Academy, Nanjing Agricultural University, Huaian, 223005 People’s Republic of China

## Abstract

**Background:**

As a key index of reproductive performance in pigs, gestation length (GL) exerts a direct influence on litter size and the survival rate of piglets. Phenotypic variability in GL has been observed across Large White pig populations. To elucidate the genetic mechanisms underlying GL, this study analyzed 22,783 reproductive records from 9,057 pigs across five Large White populations. We employed a combination of diverse analytical strategies, encompassing genome-wide association studies (GWAS), GWAS meta-analysis, Bayesian fine-mapping (BFM), transcriptome-wide association studies (TWAS), phenome-wide association studies (PheWAS), and epigenetic profiling.

**Results:**

GWAS and meta-analysis identified multiple novel GL-associated genomic regions on Sus scrofa chromosomes (SSC) 2, 7, 9, and 14. BFM refined the confidence intervals of these quantitative trait loci (QTLs), narrowing them to 10,460,892–10,737,692 bp and 31,384,086–31,384,333 bp on SSC7:, to 127,322,114–128,205,369 bp on SSC9:, and to 24,640,681–25,479,574 bp on SSC12:. TWAS revealed significant GL-related gene expression changes in relevant tissues, identifying *GNAZ* and *SLC5A4*—both located within the fine-mapped QTL on SSC14 (48.25–49.02 Mb)—as key regulators of GL. PheWAS further validated the association of *GNAZ* and *SLC5A4* with GL, while epigenetic profiling showed active chromatin states and broad promoter activity at the *GNAZ* locus, supporting its regulatory potential.

**Conclusions:**

Through multi-omics integration, this study pinpoints *GNAZ* and *SLC5A4* as core functional candidate genes regulating GL in Large White pigs. These results yield new perspectives on the genetic underpinnings governing porcine GL and supply valuable genetic materials for enhancing reproductive performance in swine breeding initiatives.

**Supplementary Information:**

The online version contains supplementary material available at 10.1186/s12711-026-01058-5.

## Background

 Gestation length (GL) is a crucial reproductive trait in pigs, directly affecting piglet survival, birth weight, and overall sow reproductive efficiency. It is typical that a GL of roughly 114 days, compared with longer or shorter gestation periods, is linked to higher birth weights and better survival outcomes in piglets [1]. However, excessively prolonged gestation can increase the risk of stillbirths, highlighting the importance of proper gestation length management for optimizing both sow productivity and piglet health. Prior research has indicated that GL is affected by both genetic and environmental elements, with maternal genetic components exerting a notable effect on its variability [2, 3].

As a classic polygenic trait, GL has been the focus of several studies that detected genetic loci associated with it [4–8]. These loci are implicated in key biological processes, encompassing hormonal regulation, placental development, and fetal growth. The final days of gestation are particularly critical, as they represent a crucial period for maturation of major fetal organs and rapid fetal weight gain. Extending GL within an optimal range can enhance fetal development, improve birth weight, and increase piglet survival rates. However, excessive prolongation may lead to higher stillbirth rates, necessitating a balanced emphasis on gestation length regulation in breeding programs.

Over the past 10 years, genome-wide association studies (GWAS) have been extensively applied to explore the genetic basis of economically vital traits in pigs, including growth [9, 10], meat quality [11, 12], reproduction [13, 14], and disease resistance [15, 16]. However, most GWAS studies rely on SNP microarrays for genotyping, which are limited by low marker density and breed-specific biases, making it challenging to capture the full spectrum of genetic variation across different populations [17]. Genotype imputation has emerged as an effective strategy to address these limitations [18, 19]. By filling in missing genotypes, this technique increases SNP density, thereby improving the power of GWAS and enabling more precise identification of trait-associated genetic variants. Furthermore, since publication of the PigGTEx project [20], integrative approaches such as transcriptome-wide association studies (TWAS) and phenome-wide association studies (PheWAS) have become increasingly accessible. These methods significantly enhance the efficiency of identifying causal genes and biological mechanisms, providing unprecedented chances to enhance our insights into the biological underpinnings of economically vital traits in pigs.

We conducted GWAS of GL in five Large White pig populations, aiming to refine the identification of genetic variants associated with this trait. We performed genotype imputation to upscale the 50 K chip dataset to whole-genome sequencing (WGS) resolution. Both pre-imputation and post-imputation datasets were utilized for conducting within-population GWAS. We also conducted a meta-analysis across populations and employed Bayesian fine-mapping (BFM) techniques to pinpoint genetic variants associated with GL in Large White pigs. Additionally, we performed TWAS and PheWAS to further prioritize putative causal genes and validate their pleiotropic effects on GL. Furthermore, by leveraging the multi-omics knowledge base Ianimal [21], we characterized the active epigenetic landscape (including ATAC-seq and histone modifications) at the candidate loci, providing direct evidence of their regulatory potential. Our results yield valuable insights into the genetic basis of GL, offering crucial genetic resources for enhancing reproductive efficiency and refining breeding strategies in swine production.

## Methods

### Animals and phenotype

Data were obtained from Large White populations from five domestic pig breeding companies in China. These pigs were introduced from Pig Improvement Company, (PIC), Dutch Topigs, Danish, French, and American strains. There were no genetic links among these five genetic strains. Rearing and performance assessment of these five strains were carried out across five farms, with one strain per farm. Between 2019 and 2024, a total of 22,783 reproductive records from 9057 pigs were collected, including 2574 from the PIC population, 1238 from the Dutch Topigs population, 2406 from the Danish population, 898 from the Frenchpopulation, and 1941 from the American population. GL was defined as the time interval (in days) between insemination and farrowing [7].

### DNA extraction, genotyping and quality control

For genotyping of the PIC population, the GeneSeek Genomic Profiler (GGP) Porcine 50 K SNP chip (Neogen) was employed. Topigs, Danish, and French populations were genotyped using the Compass Pig 50 K Plus BeadChip (Tianjin, China). Among the American population, 991 sows were genotyped using the Compass Pig 50 K Plus BeadChip, while 950 were genotyped using the 50 K liquid-phase gene chip. All SNPs were mapped to physical positions corresponding to the Sus scrofa 11.1 (Sscrofa11.1) assembly of the pig reference genome. PLINK v1.9 software [22] was adopted to conduct quality control for the obtained genotype data. Samples with an SNP call rate of less than 90% were excluded fSrom subsequent analysis. Moreover, SNPs filtering was performed separately for each population: SNPs were filtered out if they had a genotype missing rate over 0.1 or a minor allele frequency (MAF) lower than 0.05, with only SNPs situated on autosomal chromosomes kept for further analysis. Since the American Large White pigs were genotyped using two different chips, we extracted the overlapping SNPs for merging. However, after quality control, the number of retained SNPs was relatively low. Therefore, during the genotype data processing stage, the American Large White pigs were temporarily divided into two distinct groups according to their respective SNP chip platforms. In subsequent steps, genotype imputation was performed separately for each group before merging them into a unified population. After quality control, 2574 PIC sows with 38,231 eligible SNPs, 1238 Topigs sows with 36,930 eligible SNPs, 2406 Danish sows with 29,863 eligible SNPs, 898 French sows with 40,138 eligible SNPs, 950 American sows genotyped using the 50 K liquid-phase gene chip with 42,710 eligible SNPs, and 991 American sows genotyped using the Compass Pig 50 K Plus BeadChip with 38,839 eligible SNPs were used for further analysis.

### Genetic parameter estimation

We estimated the variance components and heritability for gestation length by fitting a repeatability model in DMU v6.0 [23], using the followinganimal model:

1 $$\:\mathbf{y}=\mathbf{X}\mathbf{b}+\mathbf{Z}\mathbf{a}+\mathbf{W}\mathbf{c}+\mathbf{K}\mathbf{p}\mathbf{e}+\mathbf{e}, $$

where $\:\mathbf{y}$ is the vector of GL phenotypes; $\:\mathbf{b}$ is the vector of fixed effects including year-season and parity, with season classified into four levels based on calendar months: spring (March to May), summer (June to August), autumn (September to November), and winter (December to February); $\:\mathbf{a}$ is the vector of additive genetic effects; $\:\mathbf{c}$ is the vector of random service boar effects; $\:\mathbf{p}\mathbf{e}$ is the vector of random permanent environmental effects; $\:\mathbf{e}$ is the vector of random residual effects; and $\:\mathbf{X}$, $\:\mathbf{Z}$, $\:\mathbf{W}$, and $\:\mathbf{K}$ are the incidence matrices linking $\:\mathbf{b}$, $\:\mathbf{a}$, $\:\mathbf{c},$ and $\:\mathbf{p}\mathbf{e}$ to $\:\mathbf{y}$. It was assumed that $\:\mathbf{a}$ ~ N (**0**, $\:\mathbf{A}{{\upsigma\:}}_{\mathrm{a}}^{2}$), $\:\mathbf{c}$ ~ N (**0**, $\:\mathrm{I}{{\upsigma\:}}_{\mathrm{c}}^{2}$), $\:\mathbf{p}\mathbf{e}$ ~ N (**0**, $\:\mathrm{I}{{\upsigma\:}}_{\mathrm{p}\mathrm{e}}^{2}$), and $\:\mathbf{e}$ ~ N (**0**, $\:\mathrm{I}{{\upsigma\:}}_{\mathrm{e}}^{2}$), where $\:\mathbf{A}$ is the genomic relationship matrix constructed based on SNP markers and $\:\mathrm{I}\:$is the identity matrix. The phenotypic variance ($\:{{\upsigma\:}}_{\mathrm{p}}^{2}$) was defined as the sum of $\:{{\upsigma\:}}_{\mathrm{a}}^{2}$, $\:{{\upsigma\:}}_{\mathrm{c}}^{2}$, $\:{{\upsigma\:}}_{\mathrm{p}\mathrm{e}}^{2}$, and $\:{{\upsigma\:}}_{\mathrm{e}}^{2}$. Heritability ($\:{\mathrm{h}}^{2}$) was defined as the ratio of $\:{{\upsigma\:}}_{\mathrm{A}}^{2}$ to $\:{{\upsigma\:}}_{\mathrm{p}}^{2}$.

### Genotype imputation

Our imputation strategy utilized a composite reference panel of 1662 whole-genome sequenced pigs, merging data from the PigGTEx project (1602 multi-breed pigs) [24] and a Suhuai pig cohort (*n* = 60) [25]. The workflow involved: (1) phasing the reference haplotypes using Beagle v5.2, (2) imputing the study SNP-chip data to sequencing-level density with the same tool [26, 27], and (3) applying post-imputation filters (DR² ≥ 0.9, MAF ≥ 0.05). For the American group, due to dual chip usage, we first performed genotype imputation to the whole-genome level separately for each chip dataset, and then merged the imputed datasets. The resulting high-quality imputed datasets across the five populations (PIC, Topigs, Danish, French, American) contained between 4.27 M and 6.36 M SNPs per group for subsequent association analyses. To gauge the accuracy of genotype imputation, 5% of the SNPs within the SNP chip dataset were randomly excluded, after which imputation was performed again. The allele concordance rate and correlation of the imputed and observed genotypes for these SNPs were adopted as indicators to evaluate imputation efficacy.

### GWAS

We evaluated population stratification within each of the five populations by performing independent principal component analyses (PCA) with PLINK v1.9 [22, 28]. Where stratification was detected, the top five principal components were added as covariates in the GWAS model. Genetic relationships were illustrated using FigTree v1.4.3 by constructing a neighbor-joining phylogenetic tree in MEGA6 [29] from PLINK-converted data\. The GWAS was conducted on an adjusted phenotype (individual EBV + residual from model (1)) using LDAK v5.2 [30]. Our analysis first derived a set of core SNPs using ldak-gen --thin for subsequent modeling. Association testing was then implemented using the ldak-lassosum command, utilizing a linear model and the leave one chromosome out (LOCO) method to analyze SNPs from different chromosomes separately. This approach optimizes computational resource use [31] and minimizes the loss of statistical power [32]. The linear model employed was:

2 $$\:\mathbf{y}=\mathbf{X}\mathbf{b}+\mathbf{W}\mathbf{g}+\mathbf{e}, $$

where $\:\mathbf{y}$ is the vector of the adjusted phenotypes. $\:\mathbf{b}$ is the vector of fixed effects, including the first five principal components as covariates for correction, $\:\mathbf{g}\:$is a vector of the SNP effects, $\:\mathbf{e}$ is the vector of random residual effects following $\:\boldsymbol{e}\sim\mathrm{N}(0,\mathrm{I}{{\upsigma\:}}_{\mathrm{e}}^{2})$ distribution, where $\:\mathrm{I}$ is the identity matrix, and $\:\mathbf{W}$ and $\:\mathbf{X}$ correspond to the incidence matrices of $\:\mathbf{b}$ and $\:\mathbf{g}$, respectively.

Graphical output from the GWAS (Manhattan and Q-Q plots) was produced using the CMplot package in R [33]. The Bonferroni method was applied to define significance thresholds: 0.05/N for genome-wide significance and 1/N for suggestive signals, where N is the number of quality-controlled SNPs for the chip data. Given the substantial linkage disequilibrium inherent to imputed genotypes, using the total SNP count for correction of what? is not appropriate. Consequently, for imputed data, N was set equal to the number of independent SNPs, which was estimated using PLINK v1.9 via the --indep-pairwise 50 10 0.5 function [34]. The following formula was used to estimate the proportion of phenotypic variance (PVE) attributable to each SNP:

3 $$\:\mathrm{P}\mathrm{V}\mathrm{E}\:=\:\frac{2\mathrm{p}\left(1-\mathrm{p}\right){{\upalpha\:}}^{2}}{{{\upsigma\:}}_{\mathrm{p}}^{2}},$$

where $\:\mathrm{p}$ is the minor allele frequency of the SNP, $\:{\upalpha\:}$ is the estimated effect size of the SNP, and $\:{{\upsigma\:}}_{\mathrm{p}}^{2}$ is the phenotypic variance.

### GWAS meta-analysis

The METAL software was adopted to perform a meta-analysis, for the purpose of integrating GWAS-derived data from the individual population-specific datasets [35]. The estimate of theeffect and *p*-value for each SNP were transformed into Z-score statistics. For the meta-analysis, the Z-score of each SNP was weighted and aggregated based on the sample size of the respective population, using:

4 $$\:{\mathrm{Z}}_{\mathrm{j}}\:=\:\frac{{\sum\:}_{\mathrm{i}}{{\upphi\:}}^{-1}\left(\frac{{\mathrm{P}}_{\mathrm{i}\mathrm{j}}}{2}\right)\:\times\:\:\mathrm{s}\mathrm{i}\mathrm{g}\mathrm{n}\left({{\Delta\:}}_{\mathrm{i}\mathrm{j}}\right)\:\times\:\:\sqrt{{\mathrm{N}}_{\mathrm{i}}}}{\sqrt{{\sum\:}_{\mathrm{i}}{\mathrm{N}}_{\mathrm{i}}}},$$

where $\:\mathrm{i}$ is the $\:\mathrm{i}$-th population,$\:\:\mathrm{j}$ is the $\:\mathrm{j}$-th SNP locus, $\:{{\Delta\:}}_{\mathrm{i}\mathrm{j}}\:$is the estimated effect of the $\:\mathrm{j}$-th SNP in the $\:\mathrm{i}$-th population, $\:{\mathrm{N}}_{\mathrm{i}}$ is the population size of the $\:\mathrm{i}$-th population, $\:{\upphi\:}$ represents the standard normal distribution probability density function, and $\:{\mathrm{P}}_{\mathrm{i}\mathrm{j}}$ is the *P* value of the $\:\mathrm{j}$-th SNP in the $\:\mathrm{i}$-th population. In the meta-analysis, the significance threshold was set at a suggestive level of 4.08325E-06 and a genome-wide level of 2.04162E-07.

### Bayesian fine mapping

To refine the genomic regions identified through meta-analysis, we performed Bayesian fine-mapping using the CAVIARBF (v0.2.1) method [36]. This approach leverages summary statistics (Z-scores) from the cross-population meta-analysis of imputed data, focusing on SNPs with suggestive associations within a 1-Mb window flanking the lead SNP of each significantly associated locus identified in the meta-analysis. By integrating these marginal association signals with local linkage disequilibrium (LD) patterns, CAVIARBF computes posterior inclusion probabilities to define the minimal set of variants with a 95% probability of containing the true causal mutation(s). This process yields more precise confidence intervals for QTLs, significantly improving mapping resolution [37]. The novel QTLs identified were further validated by assessing their overlap with known reproductive trait QTLs cataloged in the Pig QTL Database [38].

### TWAS and PheWAS

Transcriptome-wide association analysis was conducted using the PigGTEx TWAS server platform [20], which provides gene expression data across 34 porcine tissues (https://twas.farmgtex.org). To comprehensively assess the impact of genetically regulated gene expression on gestation length (GL), we applied the FUSION model [39, 40] to GWAS summary statistics across all available tissues. Subsequently, to validate the pleiotropic effects of the prioritized candidate genes, we performed a phenome-wide association study (PheWAS) by leveraging the PigBiobank database [41]. This resource is anchored to the Sus_scrofa_11.1 genome and provides gene-level association results for 298 production-relevant traits, categorized into six groups, including adaptation, health, and reproduction—the latter comprising 108 traits and serving as our primary focus. The *p*-values for the candidate genes were extracted from PigBiobank and used to generate a PheWAS Manhattan plot.

### Epigenetic landscape and cross-species expression profiles of candidate genes

The multi-omics knowledge base IAnimal (https://ianimal.pro/) provides access to 61,191 genomic datasets encompassing 21 species—including mouse, pig, cattle, chicken, and macaque—across multiple omics layers, including whole-genome sequencing (WGS), transcriptomics (RNA-Seq), epigenomics (ChIP-Seq, ATAC-Seq), and genomic annotations [21]. We utilized the Epigenome Signal Plotter module within IAnimal to visualize enrichment signals of ATAC-seq, H3K27ac, and H3K4me3 within the genomic regions of candidate genes. Tissue-specific mean expression levels (in TPM, Transcripts Per Million) of candidate genes in pigs and humans were obtained from the FarmGTEx TWAS-server platform (https://twas.farmgtex.org) [20].

## Results

### Descriptive statistics and heritability estimates

**Fig. 1:**
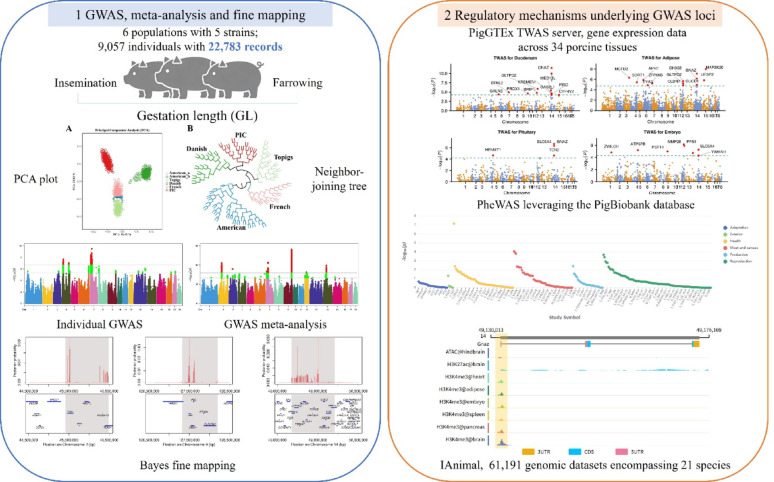
The workflows for deciphering the genetic regulatory mechanisms of gestation length trait in Large White pigs. The first layer presents information on the genome-wide association study (GWAS) population, including sample size, strain, genotype, phenotype, individual GWAS, as well as GWAS meta-analysis and Bayesian fine-mapping. The second layer describes transcriptome-wide association study (TWAS), phenome-wide association study (PheWAS), and epigenetic regulatory pattern analysis performed using public databases

**Fig. 2:**
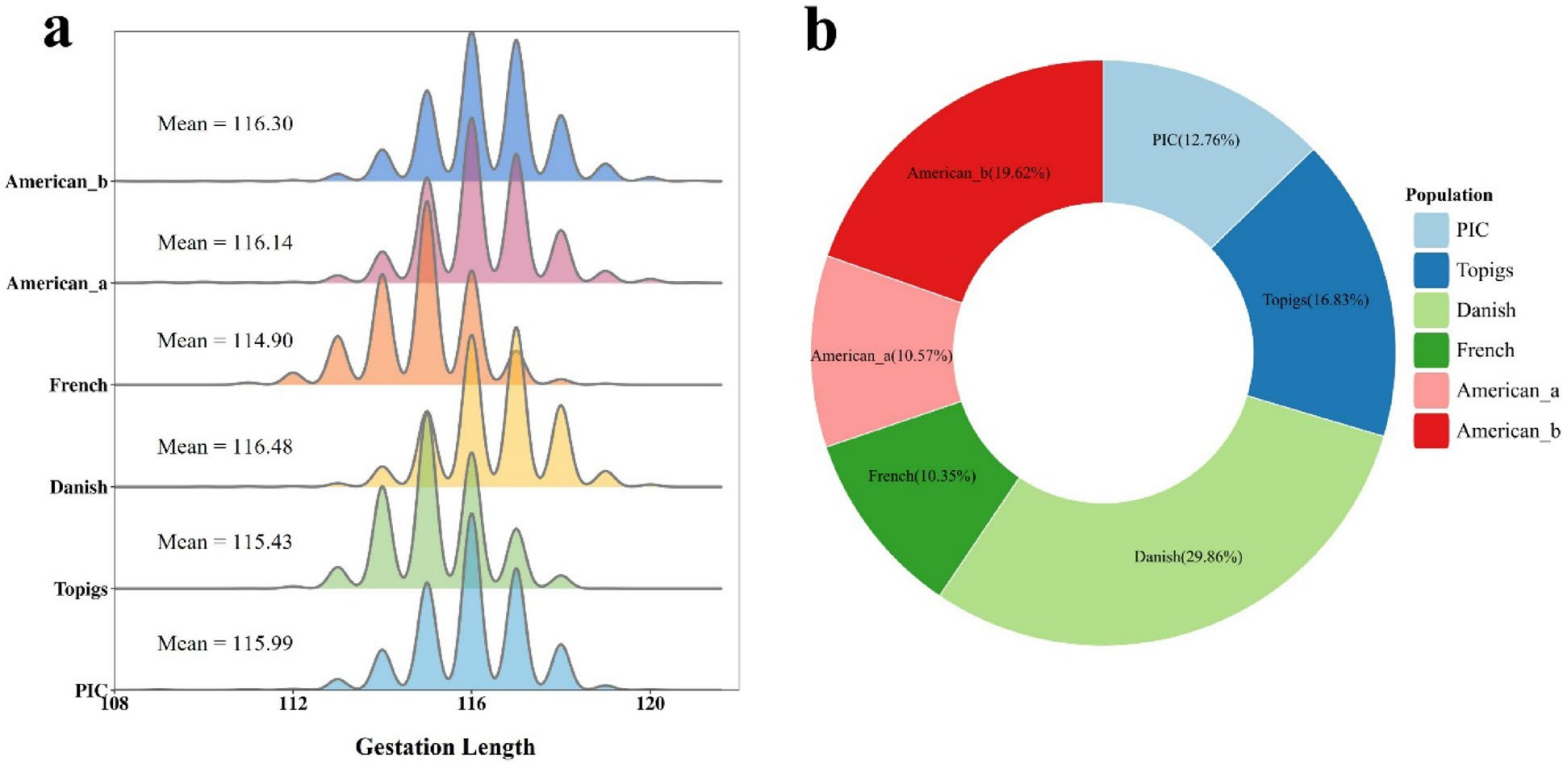
Phenotypic distribution of gestation length in six Large White pig populations. **a** Ridge plots depicting the phenotypic density distributions of gestation length across six Large White pig populations. **b** Pie charts showing the phenotypic proportions of gestation length within each of the six Large White pig populations. American_a denotes pigs genotyped with the 50 K liquid-phase gene chip, whereas American_b denotes pigs genotyped with the Compass Pig 50 K Plus BeadChip

Table 1 summarizes the descriptive statistics and heritability estimates of the GL trait across six Large White pig populations (Fig. 1). Figure 2 depicts the phenotypic distribution of the GL trait among the different populations. The Danish Large White pigs had the longest average GL, at 116.48 days. The French Large White pigs exhibited the highest heritability estimate of 0.37. Heritability estimates for the American Large White pigswere consistent at 0.30 for the two groups that were genotyped using different chips (Table 2).

**Table 1:** Descriptive statistics for gestation length in five Large White populations

Population	*N*-obs	Mean	S.D.	CV(%)	Min value	Max value	$\:\boldsymbol{h}$^2^ ± SE
PIC	2906	115.99	1.23	1.06	109	120	0.29 ± 0.03
Topigs	3835	115.43	1.14	0.99	111	119	0.17 ± 0.02
Danish	6804	116.48	1.22	1.05	110	120	0.30 ± 0.02
French	2359	114.90	1.22	1.07	110	118	0.37 ± 0.03
American_a	2408	116.14	1.37	1.18	106	121	0.30 ± 0.02
American_b	4471	116.30	1.38	1.19	106	123	0.30 ± 0.02

GL: Gestation length; N-obs: number of observations; S.D.: Standard Deviation; CV: Coefficient of variation; * h*2 ± SE: heritability ± standard error; American_a: American Large White pigs genotyped using the 50 K liquid-phase gene chip; American_b: American Large White pigs genotyped using the Compass Pig 50 K Plus BeadChip

**Table 2:** Genome-wide Association Study results for gestation length based on imputed data in five Large White populations

Population	Chr	Genomic region	*N*	Top SNP	Position	*P*- value	Var(%)	Nearest gene
PIC	4	9.66	2	rs702368253	9,660,916	6.91E-08	0.85	*ADCY8*
		13.67–14.80	16	rs341714989	14,804,675	1.59E-07	0.94	*ENSSSCG00000005971*
		16.83–17.02	142	rs344511669	16,916,396	2.20E-08	1.07	*ENSSSCG00000044389*
	7	9.41–19.05	619	rs332518905	10,583,581	3.89E-09	1.06	*ENSSSCG00000046603*
		22.72–31.41	241	rs694823388	26,232,565	3.20E-10	1.39	*FAM83B*
Topigs	11	24.46–24.48	2	rs325302738	24,483,607	1.77E-07	1.38	*TNFSF11*
Danish	2	43.18–48.28	1889	rs325456431	43,575,798	6.95E-09	0.77	*INSC*
		71.51–71.65	7	rs318295056	71,643,490	1.96E-07	0.61	*INSR*
	14	48.26–49.51	267	rs321019290	49,156,018	1.01E-07	0.85	*GNAZ*
American	8	80.44–80.45	6	rs706822629	80,444,096	1.15E-07	1.03	*NR3C2*
	12	24.47–25.47	13	rs329598694	25,479,574	9.73E-09	1.11	*ZNF652*

Chr: Sus scrofa chromosome, N: The number of significant SNPs, Position: Position of top SNP, *P*-value: p value according to the Wald test, Var: Phenotypic variation explained by the top SNP

### GWAS

**Fig. 3:**
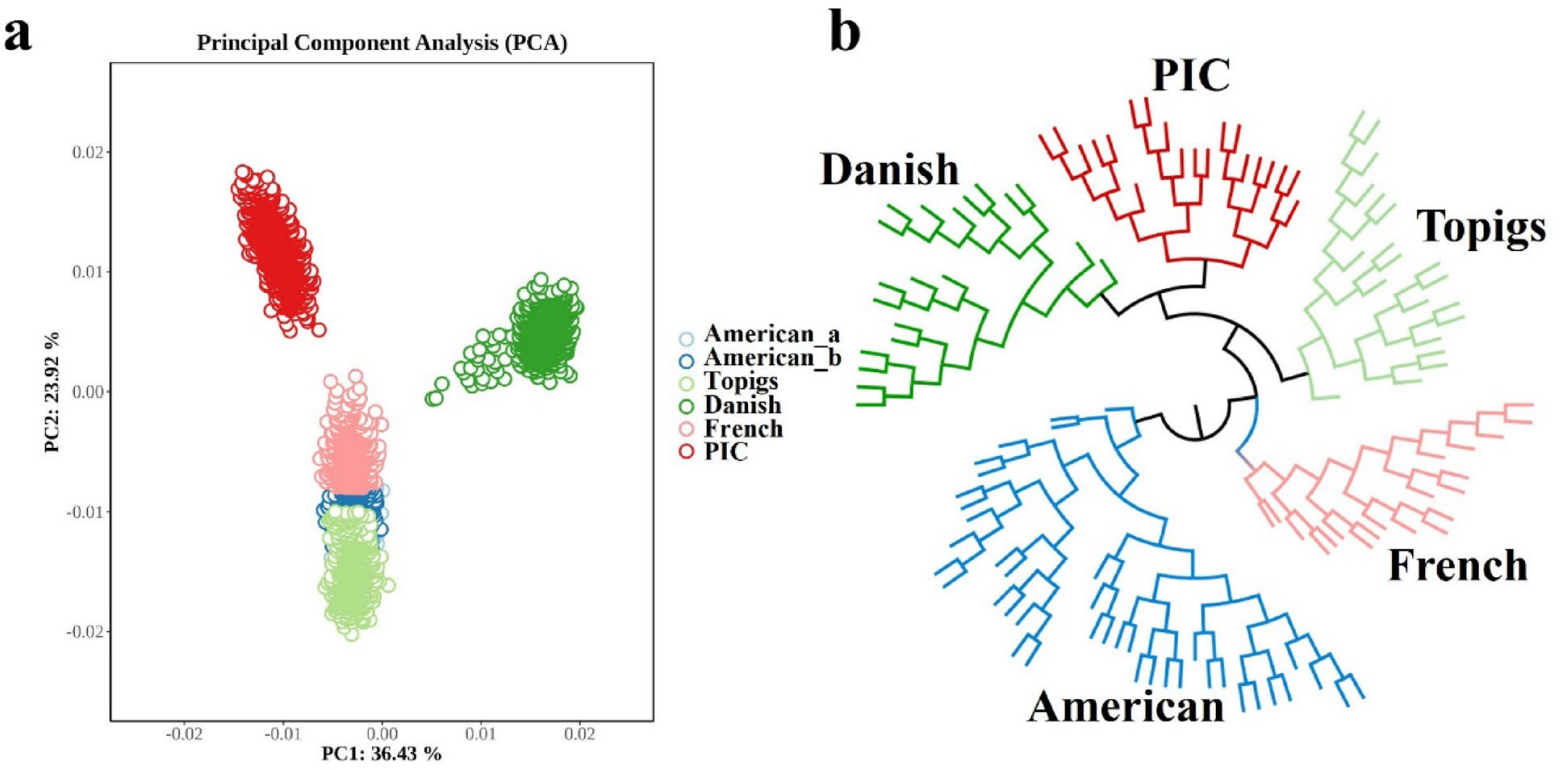
Population structure analysis of six Large White pig populations. **a** Principal component analysis (PCA) plot of population structure based on the top two principal components. PC1 and PC2 represent principal component 1 and principal component 2, respectively. **b** Neighbor-joining tree of the six Large White pig populations. American_a denotes pigs genotyped with the 50 K liquid-phase gene chip, whereas American_b denotes pigs genotyped with the Compass Pig 50 K Plus BeadChip

PCA and NJ-tree results indicated that the two American populations did not exhibit stratification. Additionally, closer genetic relationships were observed between the French Population and Topigs Population. The Danish Population exhibited slight population stratification (Fig. 3). Accordingly, in the subsequent GWAS, the top five principal components were incorporated into the model as covariates for all popuations.

**Fig. 4:**
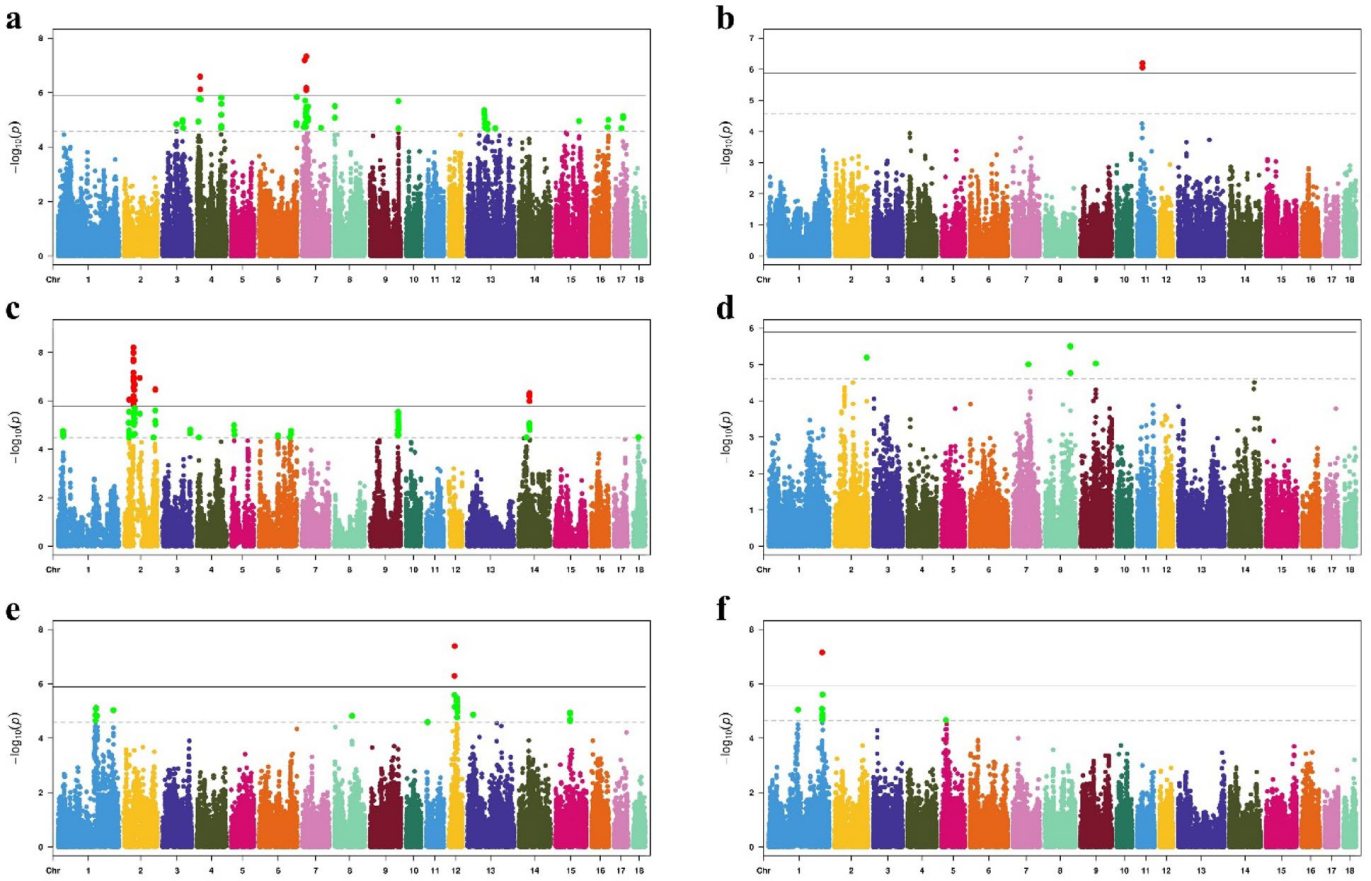
Manhattan plot of Genome-wide Association Study based on SNP chip data for gestation length in five Large White populations. **a**, **b**, **c** and **d** show the results of PIC, Topigs, Danish, and French Large White pig populations, respectively. (e) Manhattan plot for American_a (American pigs genotyped with the 50 K liquid-phase gene chip). **f** GWAS Manhattan plot for American_b (American pigs genotyped with the Compass Pig 50 K Plus BeadChip). The − log_10_-transformed *p*-value of each Single Nucleotide Polymorphisms (SNPs) is plotted versus its genomic location. Horizontal lines mark the genome-wide significance (solid) and suggestive (dashed) thresholds

**Fig. 5:**
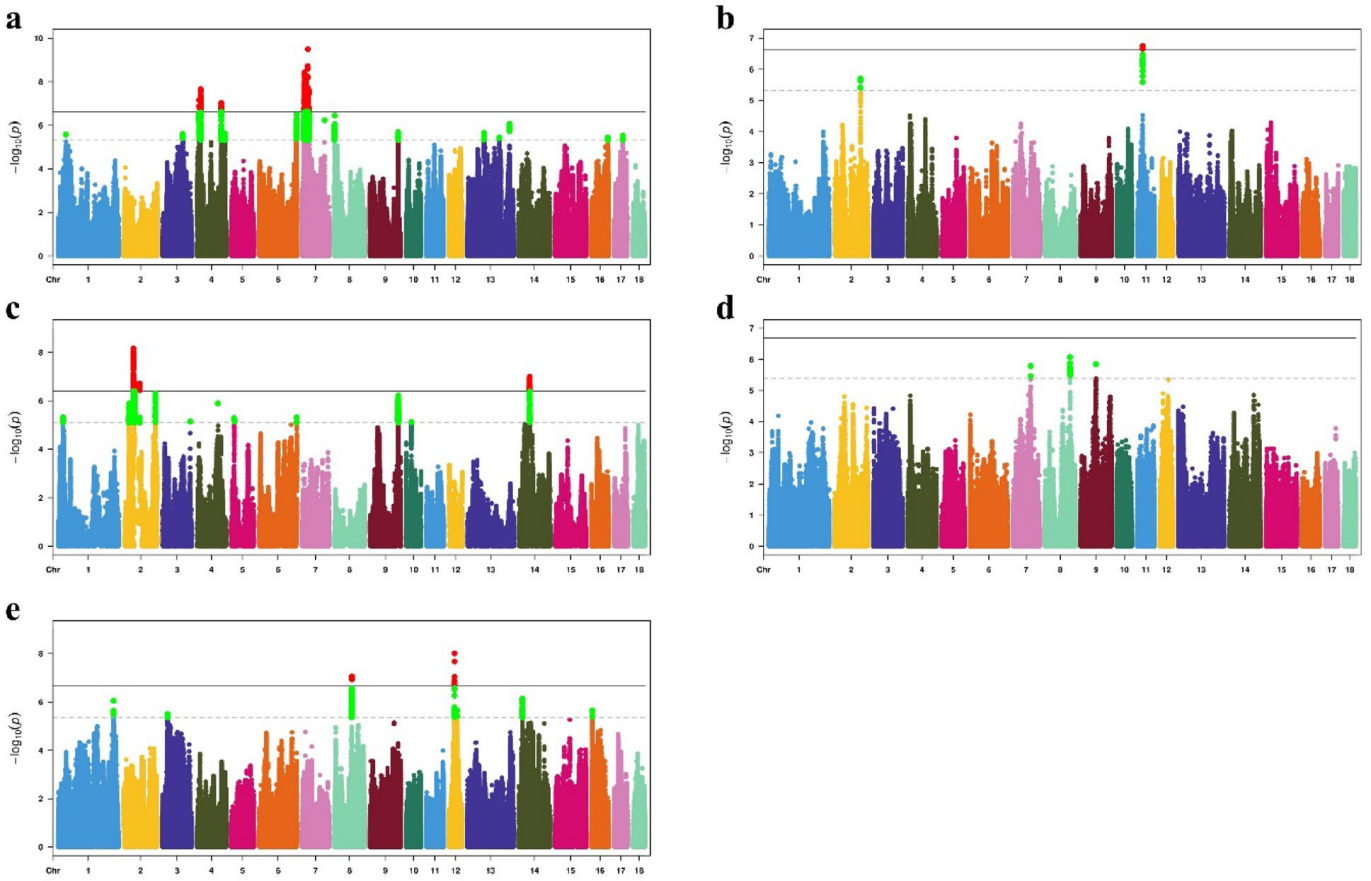
Manhattan plot of Genome-wide Association Studies based on impute data for gestation length in five Large White populations. **a**, **b**, **c**, **d** and **e** show the results of PIC, Topigs, Danish, French and American Large White pig populations, respectively. The − log_10_-transformed *p*-value of each Single Nucleotide Polymorphisms (SNPs) is plotted versus its genomic location. Horizontal lines mark the genome-wide significance (solid) and suggestive (dashed) thresholds

Among the six Large White pig populations, the mean concordance rate and correlation were greater than 97.33% and 97.81%, respectively (Additional file 2, Fig. S1). SNPs were screened according to the thresholds of MAF > 0.05 and DR^2^ > 0.9. GWAS for GL were implemented using both SNP chip and imputed whole-genome sequencing (iWGS) data. Manhattan plots corresponding to the GL trait are shown in Figs. 4 and 5, with the related Q-Q plots, as well as summary findings, shown in Additional file 2, Fig. S2, Additional file 1, Tables S1, and S2.

Based on the SNP-chip data, 62 SNPs exhibited a significant association with the GL trait in the PIC Large White pig cohort. The majority of these SNPs were mapped to SSC 3, 4, 6, and 7. The top-associated SNP, rs80812908, was positioned on SSC 7, with a *p*-value of 4.66E-08, and explained 1.0% of the phenotypic variation. For the Topigs population, the SNPs significantly linked to GL were concentrated on SSC 2; the most prominent one, rs80787622, explained 1.2% of the phenotypic variance. In the Danish population, 152 significant SNPs were detected, most of which were distributed on SSC 2, 9, and 14, with the respective counts reaching 82, 28, and 23. The most significant locus, rs81224925, was located on SSC 2, with a *p*-value of 6.27E-09. In the French population, the SNP with the strongest association, situated on SSC 9, explained as much as 1.7% of the phenotypic variance. Ultimately, in the two American populations, 43 significant SNPs in total were identified. The most significant among them, rs328501371, was located on SSC 1, with a *p*-value of 6.99E-08, and accounted for 2.3% of the phenotypic variance.

Based on the iWAS data, a GWAS analysis of the PIC population revealed 1,020 SNPs reaching genome-wide significance, with 15.7 and 84.3% located on SSC 4 and 7, respectively. The most significant SNP on SSC 7, rs694823388, had a *p*-value of 3.20E-10 and explained 1.4% of the phenotypic variation. In the Topigs population, 2 SNPs reaching genome-wide significance were observed, both located on SSC 11. No genome-wide significant SNPs were detected in the French population. The Danish population showed the largest number of genome-wide significant SNPs, totaling 2163, distributed across SSC 2 and 14. In the American population, 19 genome-wide significant SNPs were identified, with the most significant SNP, rs329598694, on SSC 12, having a *p*-value of 9.73E-09 and explaining 1.1% of the phenotypic variation.

### GWAS meta-analysis

**Fig. 6:**
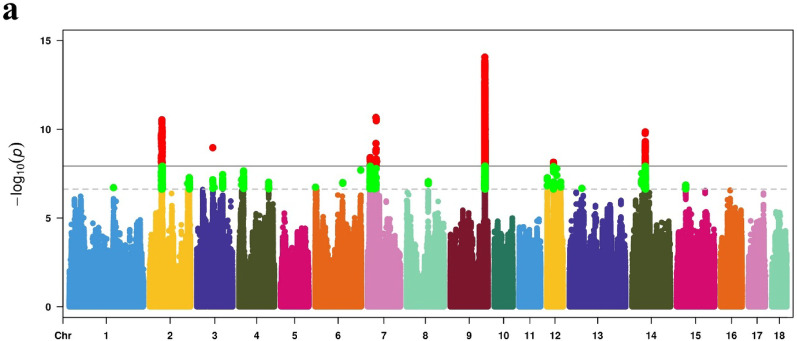
Meta-analysis results of gestation length based on imputed data across five Large White populations. **a** Manhattan plot of the meta-analysis for GL trait. The − log_10_-transformed *p*-value of each Single Nucleotide Polymorphisms (SNPs) is plotted versus its genomic location. Horizontal lines mark the genome-wide significance (solid) and suggestive (dashed) thresholds

Through a meta-analysis covering the PIC, Topigs, Danish, French, and American Large White pig populations, 3,134 SNPs with genome-wide significance were identified in total, distributed on SSC 2, 3, 7, 9, 12, and 14 (Fig. 6 and Table 3). In comparison with the GWAS findings of each independent population, the *p*-value of the most prominent SNP on SSC 2 was reduced from 6.95E-09 to 9.88E-10; the *p*-value of the most prominent SNP on SSC 7 dropped from 3.20E-10 to 2.16E-11; and the *p*-value of the most prominent SNP on SSC 14 declined from 1.01E-07 to 1.39E-10. Notably, the genomic region spanning 127.29-128.61 Mb on SSC 9 coincided with significant loci that were identified in both the PIC and Danish populations in single-population GWAS. Although the most significant SNP in this region did not initially exceed the genome-wide significance threshold, the meta-analysis significantly reduced its *p*-value to 8.43E-15. Additionally, a novel identified SNP, rs330354247, was located at 58,826,881 bp on SSC 3.

**Table 3:** Genome-wide significant meta-analysis results for gestation length based on imputed data across the five Large White populations

Chr	Genomic region	*N*	Top SNP	Position	*P*- value	Nearest gene
2	43.18–46.57	150	rs1113061053	45,509,903	2.88E-11	*FAR1*
3	58.82	1	rs330354247	58,826,881	1.08E-09	*PTCD3*
7	10.46–10.72	16	rs332518905	10,583,581	3.89E-09	*MCUR1*
	27.06–34.63	22	rs332733277	31,384,086	2.16E-11	*TCP11*
9	127.29-128.61	2707	rs326336621	127,495,052	8.43E-15	*TPR*
12	24.64–25.67	6	rs320842774	24,712,065	7.07E-09	*CDK5RAP3*
14	48.25–49.02	232	rs318823998	49,013,857	1.39E-10	*GNAZ*

Chr: Sus scrofa chromosome, N: The number of significant SNPs, Position: Position of top SNP, *P*-value: *p*-value according to the Wald test

### BFM

**Fig. 7:**
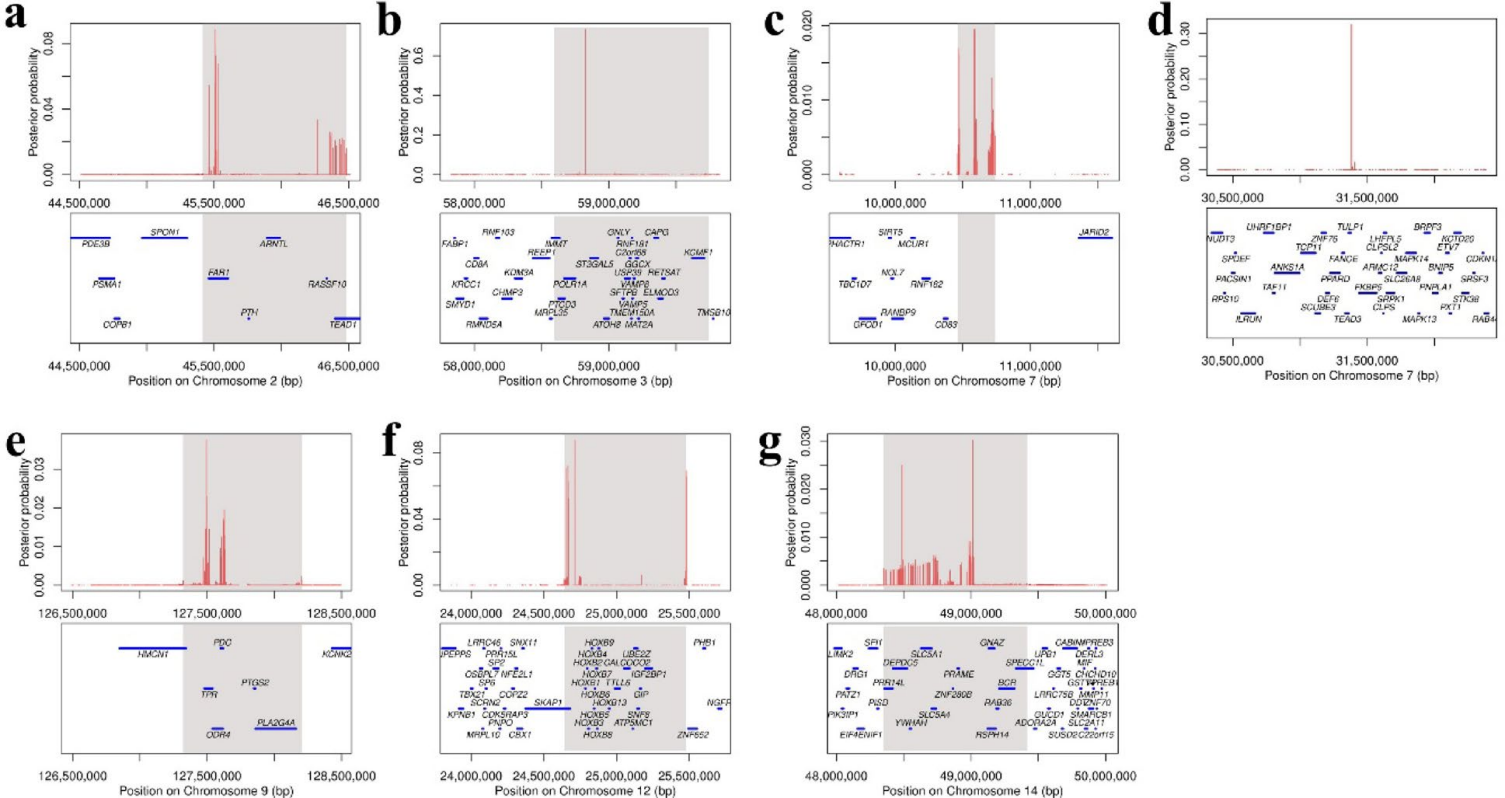
Bayes fine mapping of the Quantitative Trait Loci regulating gestation length. Bayesian fine mapping results of GL-associated QTLs on Sus scrofa Chromosome (SSC) 2 (**a**), 3 (**b**), 7 (**c**, **d**), 9 (**e**), 12 (**f**), and 14 (**g**), respectively. The x-axis represents the physical positions of Single Nucleotide Polymorphisms (SNPs) in the genome, and the y-axis indicates the posterior probability of SNPs being causal variants

The CAVIARBF method was utilized to calculate the posterior causal probability of variants within candidate regions. Causal mutations and genes were assumed to be located within a 1 Mb region upstream and downstream of the most significant SNP (Fig. 7). Bayesian fine mapping was conducted using the meta-analysis summary results to precisely localize QTLs associated with GL. The resulting 95% QTL confidence intervals were, on SSC2: 45,415,088–46,483,865 bp; on SSC3: 58,595,141–59,743,429 bp; on SSC7: 10,460,892–10,737,692 bp and 31,384,086–31,384,333 bp; on SSC9: 127,322,114–128,205,369 bp; on SSC12: 24,640,681–25,479,574 bp; and and on SSC14: 48,350,921–49,415,663 bp. Among these, the three most refined QTL confidence intervals—two on SSC7 and one on SSC12—were narrowed down to less than 1 Mb, i.e. 0.27 Mb, 0.24 kb, and 0.83 Mb, respectively.

### TWAS and PheWAS

**Fig. 8:**
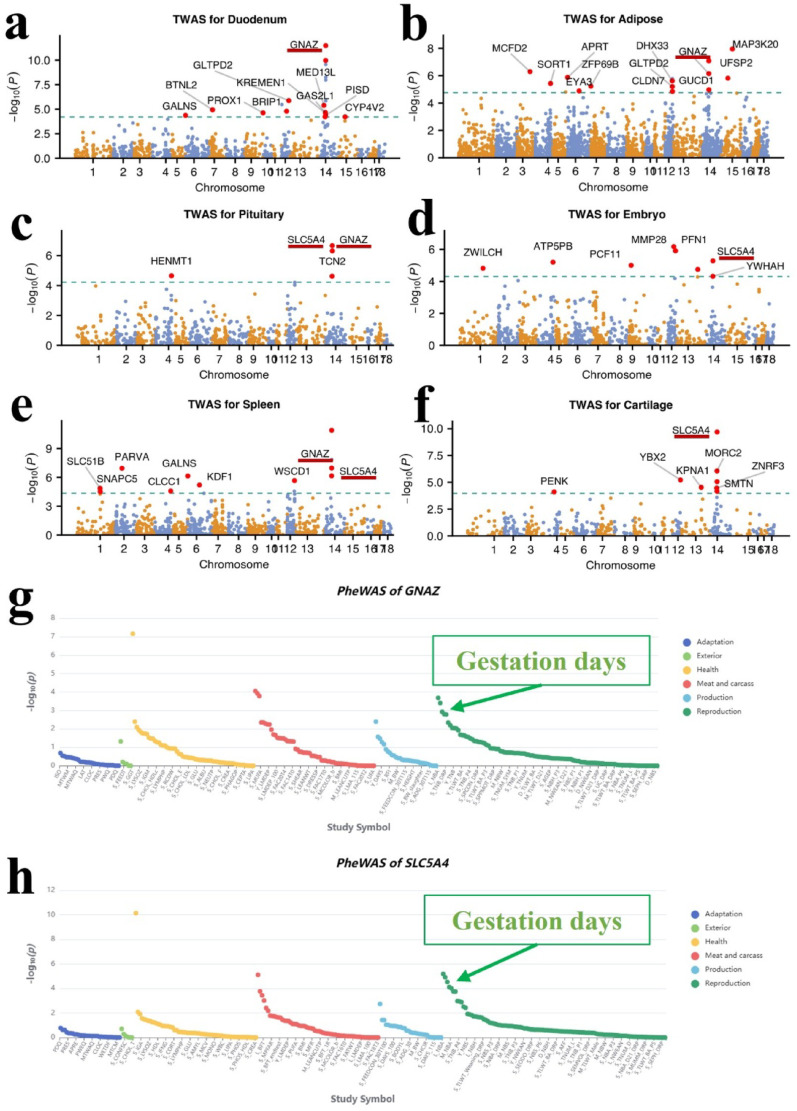
Transcriptome-wide association study and phenome-wide association study results for gestation length. Manhattan plots of TWAS between GL and gene expression levels in duodenum (**a**), adipose (**b**), pituitary (**c**), embryo (**d**), spleen (**e**), and cartilage (**f**) of Large White pigs. The x-axis represents the physical positions of genes in the genome, and the y-axis indicates − log_10_(FDR).G–H Manhattan plots of PheWAS for *GNAZ* (**g**) and *SLC5A4* (**h**) genes based on the PigBioBank database. The x-axis represents trait names, and the y-axis indicates − log_10_(*p*-value)

To validate the candidate genes identified through GWAS, we performed TWAS using gene expression data from multiple tissues sourced from the PigGTEx database. Specifically, we focused on tissues crucial for embryonic development. We compared genes that reached genome-wide significance in the TWAS analysis with those within the 95% confidence interval obtained from Bayesian fine-mapping. These analyses identified *GNAZ* and *SLC5A4* —both located within the key SSC14 QTL (48.25–49.02 Mb)—as significant candidates in both TWAS and GWAS. The expression levels of *GNAZ* in the duodenum, spleen, pituitary, and adipose tissues were significantly associated with the GL trait in Large White pigs (*p* < 7.09E-07). Similarly, the expression levels of *SLC5A4* in cartilage, pituitary, spleen, and embryo tissues also demonstrated significant associations with the GL trait (*p* < 5.15E-06). Subsequently, we conducted PheWAS on *GNAZ* and *SLC5A4* using the PigbioBank database. The results demonstrated that both these genes were significantly associated with the GL trait in pigs (*p* < 0.01) (Fig. 8 and Additional file 1, Table S2). In conclusion, *GNAZ* and *SLC5A4* were identified as potential functional candidate genes for the GL trait in Large White pigs.

### Epigenetic landscape and cross-species expression profiles of candidate genes

**Fig. 9:**
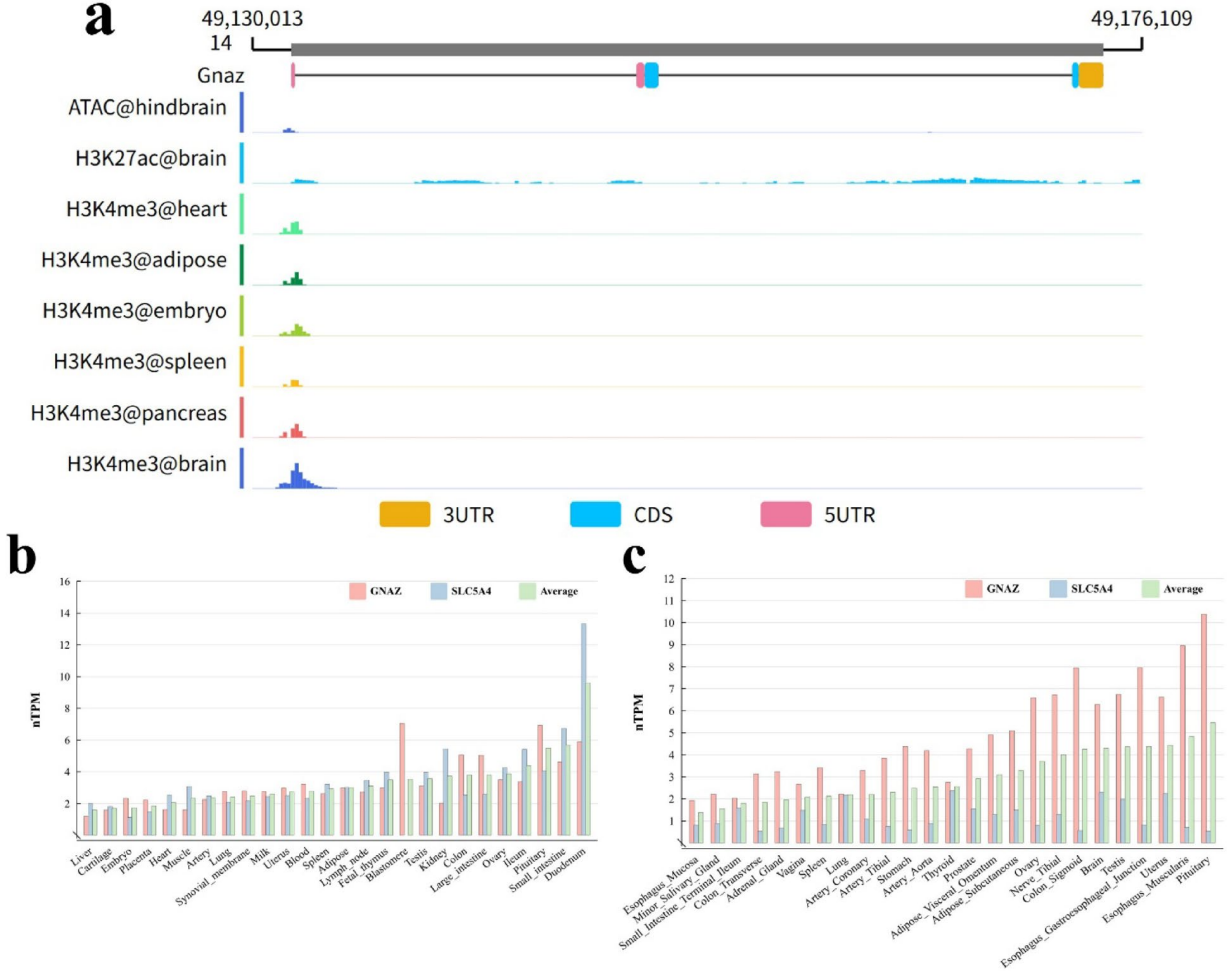
Multi-species functional genomic annotation and expression profiles of *GNAZ* and *SLC5A4*. **a** Epigenetic landscape of the *GNAZ* locus (Sus scrofa chr14:49,130,013–49,176,109 bp). From top to bottom: ATAC-seq signal reflecting open chromatin regions in hindbrain tissue; H3K27ac ChIP-seq signal marking active enhancers and promoters in brain tissue; H3K4me3 ChIP-seq signal (a hallmark of active promoters) across six tissues (heart, adipose, embryo, spleen, pancreas, and brain); and the *GNAZ* gene model (gray) at the bottom, with 5’UTR, coding sequences (CDS), and 3’UTR labeled. **b** Mean expression levels (Transcripts Per Million, TPM) of *GNAZ* and *SLC5A4* across various tissues from the PigGTEx database. **c** Mean expression levels (TPM) of *GNAZ* and *SLC5A4* across homologous tissues from the human database, facilitating cross-species comparison

To elucidate the potential regulatory mechanisms of the QTL on SSC14, we systematically investigated the epigenetic features of the *GNAZ* locus (chr14:49,130,013–49,176,109) in pigs (Sus scrofa) (Fig. 9a). ATAC-seq peaks in the hindbrain revealed pronounced chromatin accessibility near the promoter region. Consistent with this, H3K27ac signals in brain tissue overlapped with these accessible regions, suggesting active enhancer or promoter states. Furthermore, H3K4me3—a canonical mark of active promoters—was detectable across six examined tissues (heart, adipose, embryo, spleen, pancreas, and brain), indicating basal transcriptional activity of *GNAZ* in diverse biological contexts. In contrast, significant open chromatin signals were not detected for *SLC5A4* in the available datasets.

We next compared the expression patterns of *GNAZ* and *SLC5A4* across multiple species. Transcriptomic data from 26 pig tissues demonstrated that *GNAZ* is moderately yet broadly expressed. Both genes showed relatively high expression levels in intestinal and pituitary tissues (Fig. 9b). Expression profiles from 25 human tissues in the GTEx database recapitulated this pattern: *GNAZ* was widely expressed, and both genes were highly expressed in the pituitary gland (Fig. 9c). This cross-species conservation in expression suggests that *GNAZ* and *SLC5A4* may play important and evolutionarily conserved roles in neuroendocrine or metabolic regulation.

## Discussion

### Heritability estimates

Heritability of GL was estimated in this study using a repeatability model, with estimates ranging from 0.17 to 0.37 across the five Large White pig populations. Previous studies have consistently indicated that GL is a moderately heritable trait [42]. For instance, heritability estimates were 0.25–0.29 in Dutch Landrace pigs [43], 0.29 in Landrace pigs, and 0.34 in Yorkshire pigs [44]. In mixed populations of Large White, Landrace, and Duroc pigs, heritability was estimated at 0.16 [7]. Similarly, in Large White pigs, heritability was reported as 0.16 [45]. In Hungarian Landrace pigs, heritability was 0.18, while in Hungarian Large White pigs, it was 0.26 [46]. Furthermore, in a mixed population of Large White and Landrace pigs, heritability was estimated at 0.21 [47]. The heritability estimates of GL in this study align with previous findings, confirming that GL is a heritable trait and establishing a foundation for future genomic selection to enhance phenotypic performance.

### GWAS on GL traits

In recent years, the rapid advancement of sequencing technologies and the growing demand for high-density markers have led to the widespread application of genotype imputation in genetic research. Genotype imputation enables the projection of low-density marker chip data onto iWGS data, thereby significantly expanding the range of markers available for association analysis. Nevertheless, the accuracy of imputation is influenced by multiple factors, such as the density of target SNPs, the size of the reference population, the genetic distance between the target and reference populations, and the algorithms employed in the imputation process [48]. In this study, we utilized the Beagle software to impute SNP chip data to iWGS data, achieving an imputation accuracy exceeding 97.33%. This high level of accuracy establishes a robust foundation for subsequent GWAS analysis, ensuring the reliability and validity of the results.

In the GWAS of GL in Large White pigs, we identified multiple significant genomic regions associated with GL. Notably, QTLs related to GL were previously reported on several pig chromosomes, including SSC1, SSC2, SSC5, and SSC14. In this study, leveraging imputed data, we detected a significant region in Danish Large White pigs (SSC1: 43.18–48.28 Mb) that exhibits substantial overlap with the region identified using the PorcineSNP60 BeadChip and Bayesian statistical methods in commercial sows [49], thereby validating the importance of these regions for GL. Moreover, the significant region identified on SSC15 (24.47–25.47 Mb) aligns with the region determined through integrated GWAS analysis of multiple breeds in the PigGTEx database, suggesting conserved genetic effects across breeds [50]. Additionally, the significant SNP identified on SSC12 in Topigs Large White pigs is located within 0.2 Mb of the SNP rs80949037 previously reported to be associated with GL in Large White pigs [5], further supporting the potential presence of functional genes related to GL in these regions. Beyond regions overlapping or closely related to existing studies, this study also revealed several novel significant genomic regions, which may offer new insights into the genetic basis of GL traits in Large White pigs.

### Multi-population meta-analysis

The statistical power of single-population GWAS is frequently constrained by limited sample sizes, whereas meta-analysis of GWAS summary statistics (Meta-GWAS) offers a robust approach to substantially enhance statistical power [51, 52]. Meta-GWAS integrates GWAS summary statistics from multiple populations, effectively enlarging the sample size and thereby improving the detection of regions significantly associated with gestation length traits in Large White pigs. However, Meta-GWAS is not without challenges, including discrepancies in available SNPs across populations, which often reduce the number of SNPs shared among all populations, complicating the effective execution of GWAS [53]. In this study, we addressed this issue by employing a comprehensive pig genotype imputation reference panel to standardize SNP profiles across diverse populations. Through Meta-GWAS analysis of five Large White pig populations, we identified 3,134 SNPs that exceeded the genome-wide significance threshold, including the novel SNP rs330354247 (SSC3:58826881). In single-population GWAS using imputed data, the most significant SNP was rs694823388 on SSCe 7 in the PIC Large White population, with a *p*-value of 3.20E-10. In contrast, in the Meta-GWAS the most significant SNP (SSC9:127495052) had a lower *p*-value of 8.43E-15. Notably, this SNP surpassed the significance threshold only in the PIC and Danish Large White populations in single-population GWAS. This demonstrates that Meta-GWAS can validate the robustness of GWAS results across diverse populations, facilitating the identification of consistent genetic signals [54, 55].

However, we also observed cases where regions significant in single-population GWAS showed diminished signals in the Meta-GWAS. For instance, a genome-wide significant region on SSC 4 identified in the PIC Large White single-population GWAS exhibited reduced signal strength in the Meta-GWAS. This may result from differences in genetic backgrounds, genetic effects, and environmental factors among populations, introducing heterogeneity in the Meta-GWAS analysis [56].

### BFM

The Bayesian fine mapping method (CAVIARBF) is based on a Bayesian statistical framework and facilitates the precise localization of causal variants by integrating marginal test statistics with linkage disequilibrium (LD) information between SNPs [36]. The core of this method lies in calculating the posterior inclusion probability (PIP) for each SNP as a causal variant, thus enhancing the accuracy of identifying potential variants [57]. Compared to traditional *p*-value-based methods, CAVIARBF not only accounts for the marginal effects of SNPs but also incorporates LD information between SNPs, allowing for a more comprehensive evaluation of the probability that a SNP is a causal variant. In this study, we applied the CAVIARBF method to perform fine mapping analysis using Meta-GWAS data for GL in Large White pigs. Through this analysis, we identified the 95% confidence intervals for the seven most significant QTLs, on SSC2, SSC3, SSC7, SSC9, SSC12, and SSC14 in Large White pigs. Specifically, the two QTL intervals on SSC7 were 10,460,892–10,737,692 bp and 31,384,086–31,384,333 bp; the QTL interval on SSC9 was 127,322,114–128,205,369 bp; and the QTL interval on SSC12 was estimated to be 24,640,681–25,479,574 bp. Notably, the ranges of these four QTL intervals were successfully narrowed down to less than 0.88 Mb. By refining the QTL intervals, we achieved a more precise localization of potential genetic variants, thereby offering robust support for elucidating the genetic basis of GL traits.

### TWAS and PheWAS

In the field of porcine reproductive trait research, GWAS have successfully identified several putative functional candidate genes associated with GL, including HUNK [7], TROAP [8], and ADCY5 [45]. These investigations have provided fundamental insights into the genetic architecture underlying GL variation in swine. However, conventional GWAS approaches exhibit two principal limitations when characterizing genomic loci influencing complex traits: (1) LD obscures the discrimination between truly causal variants and correlated signals, and (2) the methodology cannot directly elucidate the mechanistic pathways through which genetic variants influence phenotypic expression through gene regulatory networks [58]. Consequently, while GWAS effectively identifies trait-associated genomic regions, the precise determination of causal genes and their functional biological mechanisms remains methodologically challenging. TWAS has emerged as a powerful methodological framework for inferring putative causal relationships between gene expression patterns and complex phenotypic traits. This approach integrates expression QTL (eQTL) reference panels—comprising both transcriptomic and genotypic data—with GWAS datasets to identify robust gene-trait associations [59]. The methodological foundation of TWAS involves the prediction of individual gene expression levels based on genetic data, followed by association testing between these predicted expression values and phenotypic measurements to identify potentially causal genes. In the current investigation, we implemented an integrative multi-omics approach combining TWAS with PheWAS to systematically validate GWAS-identified candidate genes. Specifically, we employed comprehensive multi-tissue gene expression data from the PigGTEx database, with particular emphasis on tissues biologically relevant to embryonic development, including embryonic and pituitary tissues. Through TWAS analysis, we found that the expression levels of *GNAZ* in the duodenum, spleen, pituitary, and adipose tissues were significantly associated with the GL trait in Large White pigs. Additionally, the expression levels of *SLC5A4* in cartilage, pituitary, spleen, and embryo tissues demonstrated significant associations with the GL trait. *GNAZ* is involved in cellular signal transduction, influencing cell growth and division through the MAPK/ERK pathway, thus playing a role in embryonic development and tissue homeostasis [60]. Moreover, *GNAZ* regulates calcium signaling and PKC activity in immune cells, which may affect maternal immune tolerance during pregnancy [61]. *SLC5A4* plays a crucial role in maintaining blood glucose levels and energy metabolism, contributing to maternal glucose homeostasis during pregnancy [62]. These findings suggest that *GNAZ* and *SLC5A4* may influence pig GL traits through their expression regulation in specific tissues. Subsequently, we conducted PheWAS analysis using the PigbioBank database to further confirm the association of these two genes with the GL trait. PheWAS, by analyzing the association between genes and multiple phenotypes, reveals gene pleiotropy, providing more comprehensive evidence for gene function validation. The multi-omics data integration approach adopted in this study not only improved the accuracy of candidate gene identification but also provided stronger evidence for subsequent functional validation.

### Epigenetic landscape and cross-species expression profiles of candidate genes

Epigenetic regulation refers to a suite of heritable mechanisms that modulate gene expression without changing the underlying DNA sequence [63]. This layer of control, which includes processes such as histone modification and chromatin remodeling, is essential for coordinating gene activity during development and in response to environmental stimuli [64]. The epigenetic profiling of the *GNAZ* locus revealed signatures of active regulation across multiple porcine tissues. The presence of open chromatin (ATAC-seq), enhancer marks (H3K27ac), and active promoter marks (H3K4me3) in key developmental and endocrine tissues is consistent with the TWAS results, which showed significant associations between *GNAZ* expression in the pituitary, adipose, and other tissues and GL. This convergence of epigenetic activity and TWAS signals suggests that *GNAZ* expression is tightly regulated in a tissue-specific manner, and its modulation could indeed influence the physiological processes governing gestation. Furthermore, the observed cross-species conservation in expression profiles—with both *GNAZ* and *SLC5A4* showing high expression in the pituitary and intestinal tissues in both pigs and humans—strengthens the case for their fundamental role in neuroendocrine or metabolic pathways that are critical for pregnancy maintenance and timing. The pituitary gland, in particular, is a master regulator of hormonal secretion; the high expression of both genes here aligns perfectly with the known importance of hormonal control in parturition timing. The PheWAS results, which confirmed a significant association between these genes and GL, alongside associations with other traitsamong other traits, further underscore their pleiotropic effects and central biological importance.

## Conclusions

This study utilized a comprehensive approach integrating GWAS, meta-analysis, Bayesian fine mapping, TWAS, and PheWAS to systematically investigate the genetic basis of GL in Large White pigs. By analyzing 9057 pigs from five populations, we identified multiple novel QTL regions significantly associated with GL, the significance of which was further validated through multi-population meta-analysis. The application of Bayesian fine mapping refined the confidence intervals of the QTLs, TWAS and PheWAS analyses revealed significant associations of *GNAZ* and *SLC5A4* within the refined QTL region with GL. Our multi-omics approach seamlessly integrated statistical genetics (GWAS, TWAS, PheWAS) with functional genomics (epigenetic annotation and expression conservation). The concordance of these independent lines of evidence firmly established *GNAZ* and *SLC5A4* as important and plausible regulators of embryonic development and gestation length in pigs, likely through mechanisms involving hormone signaling and energy metabolism.

## Supplementary Information

Below is the link to the electronic supplementary material.

Supplementary Material 1

Supplementary Material 2

## Data Availability

The phenotype data and SNP-chip data used in the present study are deposited in the figshare repository (10.6084/m9.figshare.30846728).
