# Supplementary material for: Integrative multi-omics analysis identifies GNAZ and SLC5A4 as critical regulators of gestation length in Large White pigs
Source: Genet Sel Evol. 2026 Jun 10;58:41. doi: 10.1186/s12711-026-01058-5 (PMC13479978; doi:10.1186/s12711-026-01058-5)
Supplement: Supplementary file 1 [file 12711_2026_1058_MOESM1_ESM.docx]

Table S1 **Genome-wide Association Study (GWAS) results for gestation length (GL) traits based on chip data.**

| **Population** | **Chr** | **N** | **Lead SNP** | **Position (bp)** | ***P* value** | **Var (%)** |
| --- | --- | --- | --- | --- | --- | --- |
| PIC | 3 | 4 | rs81242056 | 90753933 | 1.01E-05 | 0.76 |
|  | 4 | 10 | rs80985215 | 14332353 | 2.58E-07 | 0.87 |
|  | 6 | 3 | rs81273774 | 168931165 | 1.45E-06 | 0.77 |
|  | 7 | 23 | rs80812908 | 19010124 | 4.66E-08 | 1.04 |
|  | 8 | 2 | rs340032616 | 2843234 | 3.15E-06 | 0.66 |
|  | 9 | 2 | rs81300514 | 127551686 | 2.04E-06 | 0.74 |
|  | 13 | 11 | rs81446757 | 74697976 | 4.46E-06 | 0.77 |
|  | 15 | 1 | rs81301356 | 106599290 | 1.10E-05 | 0.63 |
|  | 16 | 2 | rs81463487 | 75717226 | 9.94E-06 | 0.64 |
|  | 17 | 4 | rs330730198 | 40222297 | 7.22E-06 | 0.59 |
| Topigs | 11 | 2 | rs80787622 | 24468975 | 8.76E-07 | 1.23 |
| Danish | 1 | 6 | rs80991420 | 22853943 | 1.73E-05 | 0.46 |
|  | 2 | 83 | rs81224925 | 43735602 | 6.27E-09 | 0.79 |
|  | 3 | 2 | rs81235137 | 125353696 | 1.57E-05 | 0.44 |
|  | 4 | 1 | rs80797146 | 8926615 | 3.28E-05 | 0.41 |
|  | 5 | 3 | rs80851822 | 13913761 | 1.03E-05 | 0.57 |
|  | 6 | 5 | rs345072152 | 144294711 | 1.73E-05 | 0.54 |
|  | 9 | 28 | rs81417434 | 126718858 | 2.85E-06 | 0.55 |
|  | 14 | 23 | rs332196848 | 49348205 | 4.77E-07 | 0.78 |
|  | 18 | 1 | rs81297245 | 25220720 | 3.20E-05 | 0.44 |
| French | 2 | 1 | rs81304138 | 141877566 | 6.43E-06 | 1.51 |
|  | 7 | 1 | rs319872961 | 70169458 | 9.88E-06 | 1.46 |
|  | 8 | 3 | rs323548624 | 114808210 | 3.06E-06 | 1.62 |
|  | 9 | 1 | rs81308335 | 125722055 | 9.33E-06 | 1.65 |
| American_a | 1 | 10 | rs328501371 | 241533195 | 6.99E-08 | 2.34 |
|  | 5 | 1 | rs80838995 | 19697604 | 2.15E-05 | 1.47 |
| American_b | 1 | 9 | rs342387771 | 171487562 | 7.65E-06 | 1.72 |
|  | 8 | 1 | rs327302179 | 80472142 | 1.49E-05 | 1.32 |
|  | 11 | 1 | rs81304673 | 4705542 | 2.55E-05 | 1.00 |
|  | 12 | 14 | rs81342863 | 25478368 | 4.09E-08 | 1.68 |
|  | 13 | 1 | rs81443841 | 24818022 | 1.36E-05 | 1.24 |
|  | 15 | 6 | rs345265741 | 66267649 | 1.14E-05 | 1.00 |

Chr: *Sus scrofa* chromosome, N: The number of significant SNPs, Position: Position of top SNP, *P*-value: *p* value according to the Wald test, Var: Phenotypic variation explained by the top SNP. American_a: American Large White pigs genotyped using the 50K liquid-phase gene chip; American_b: American Large White pigs genotyped using the Compass Porcine 50K Plus breeding chip.

Table S2 **Transcriptome-wide association study (TWAS) results for gestation length (GL) traits.**

| **GeneName** | **CHR** | **Start** | **End** | **TWAS.P** | **TWAS.Z** | **FDR** | **GeneName.tissue** |
| --- | --- | --- | --- | --- | --- | --- | --- |
| *GNAZ* | 14 | 49132013 | 49174109 | 3.38E-12 | -6.960895704 | 2.72E-09 | GNAZ:Duodenum |
|  | 14 | 49132013 | 49174109 | 1.06E-07 | -5.315922892 | 3.14E-05 | GNAZ:Spleen |
|  | 14 | 49132013 | 49174109 | 4.84E-07 | -5.032616467 | 2.00E-04 | GNAZ:Pituitary |
|  | 14 | 49132013 | 49174109 | 7.09E-07 | -4.958846344 | 5.09E-04 | GNAZ:Adipose |
| *SLC5A4* | 14 | 48708047 | 48741440 | 2.04E-10 | 6.358212138 | 9.49E-08 | SLC5A4:Cartilage |
|  | 14 | 48708047 | 48741440 | 2.15E-07 | 5.185681559 | 1.78E-04 | SLC5A4:Pituitary |
|  | 14 | 48708047 | 48741440 | 6.97E-07 | 4.962234959 | 1.34E-04 | SLC5A4:Spleen |
|  | 14 | 48708047 | 48741440 | 5.15E-06 | 4.558423409 | 1.59E-03 | SLC5A4:Embryo |

Chr: *Sus scrofa* chromosome, Start: the start position of the gene, End: the end position of the gene
