# Supplementary material for: Integrative multi-omics analysis identifies GNAZ and SLC5A4 as critical regulators of gestation length in Large White pigs
Source: Genet Sel Evol. 2026 Jun 10;58:41. doi: 10.1186/s12711-026-01058-5 (PMC13479978; doi:10.1186/s12711-026-01058-5)
Supplement: Supplementary file 2 [file 12711_2026_1058_MOESM2_ESM.docx]

**Figure S1. Quality Assessment of Chip Data Imputed to Whole-Genome Resequencing Level.**

The average consistency rate (A) and correlation (B) of imputation to whole-genome sequence by chromosome. The average accuracy of imputation shows in histogram before (blue) and after (red) filtering. American_a represents the American Large White pigs genotyped using the 50K liquid-phase gene chip, while American_b represents the American Large White pigs genotyped using the Compass Porcine 50K Plus breeding chip.

Figure S2. **Quantile-quantile plot of gestation length (GL) for Large White pigs.**

(A) Quantile-quantile plot of GWAS results based on chip data, (B) Quantile-quantile plot of GWAS results based on impute data.
